# Kindness Is the Language That the Deaf Can Hear and the Blind Can See: Kindness, Theory of Mind and Well-Being in Adolescents

**DOI:** 10.3390/children11121555

**Published:** 2024-12-21

**Authors:** Poppy Stamp, Sandra Bosacki, Victoria Talwar

**Affiliations:** 1 Department of Psychology, University of Exeter, Stocker Rd., Exeter EX4 4PY, UK; 2 Faculty of Educational Studies, Brock University, 1812 Sir Isaac Brock Way, St. Catharines, ON L2S 3A1, Canada; sbosacki@brocku.ca; 3 Department of Educational and Counselling Psychology, McGill University, Education Building, 3700 Mc Tavish St., Montreal, QC H3A 1Y2, Canada; victoria.talwar@mcgill.ca

**Keywords:** theory of mind, kindness, adolescence, well-being, compassion

## Abstract

Background/Objectives: This mixed-methods, cross-sectional study explored adolescent understandings of kindness, and interconnections amongst Theory of Mind (ToM; ability to attribute mental states to oneself and others), kindness, compassion, and social-psychological well-being components in 318 participants aged 10–18 (Mage = 14.58, *SD* = 2.31). Methods: Participants completed a battery of self-report measures and wrote responses to open-ended questions about kindness in different relational and situational contexts. Results: Most adolescents gave other-oriented, psychological definitions of kindness, increasing in detail with age. Content analysis revealed main themes of helping, followed by proactive support and respect, and differed according to the identity of the recipient (stranger, self, others), and situational context (home, school). Results showed significant positive correlations between perceptions of kindness and social (but not psychological) well-being, with gender and age differences. High levels of ToM related to high levels of perceived compassion and kindness for others, and the relations strengthened with age. Conclusions: The results highlight implications for future research on adolescents’ perceptions of prosociality, and kindness-based mental health interventions that promote social cognition and prosocial acts.

## 1. Introduction

### 1.1. Definitions of Kindness

What does it mean to be kind? In the psychological literature, there is considerable variation when answering this question. Some argue that kindness is simple and requires low effort [1], whereas others view it as high-cost and altruistic [2,3]. Alternatively, some studies focus on the emotional, internal motivation behind kindness as its defining factor. For example, Canter et al. [4] suggest that kindness is not just its associated actions, but an inclination to act with “a genuine warm feeling for others”. Notably, most studies focus on kindness as an other-oriented, prosocial phenomenon [5,6]; however, it has been also considered a way of being towards oneself, especially in challenging times, that is closely related to self-compassion [7,8]. The present study chose to explore kindness in two ways: firstly, we explored how youth conceptualised kindness in their own terms, and secondly, we measured how kindness (defined as an inclination to engage in behaviours contributing to others’ welfare [9]) was linked to well-being and certain cognitive traits.

### 1.2. Kindness in Adolescents

To the best of our knowledge, to date there remain few studies specifically exploring adolescents’ own definitions of kindness and how this develops with age. In one study of 11–15-year-olds, Cotney and Banerjee [10] asked: ‘What does kindness mean to you?’. Their results identified the themes of emotional support, helping, generosity, honesty, forgiveness, compliments, proactive support, formal kindness and social inclusion. A 2019 study by Binfet [11] asked children to execute kind acts, which replicated previously emergent themes of helping, giving and being respectful. However, such studies focused on children below 15 years of age—developmental research that samples the full adolescent age-range remains sparse. Building on these studies, the present study explores the conceptualisation of kindness in an adolescent sample aged between 10 and 18 years. Through this, we hope to explore developmental trends in kindness conceptualisation, thereby filling a gap in the previous literature.

The majority of past studies show that the most common recipients of kindness are those that are familiar to the actor, such as friends, peers or family [10,11,12,13], when studying an adult sample. These results suggest that it would be beneficial for research to further explore the adolescent-specific differences in kindness towards those who are relationally close versus those who are not (i.e., strangers). Further exploration of how the recipient’s relationship to the actor in this age group may influence kindness may inform anti-bullying interventions by informing educators on how to encourage kindness towards socially isolated pupils.

### 1.3. Theory of Mind and Kindness

Research on adolescents’ perceptions of kindness is particularly valuable as, in that period, they are in the midst of a critical period of social-cognitive development. The neurotypical adolescent brain is characterised by the redevelopment of perspective-taking regions, leading to more complex relational understandings [14]. Specifically, this includes increasingly abstract understandings of mental states belonging to the self and others [15], which is a progression from childhood experiences of understanding basic emotions and false beliefs [16]. With regards to gender differences in adolescent cognitive empathy, there are mixed findings. Some studies have found that girls are more advanced than boys in perspective-taking [17,18] while others have reported no gender differences [19]. These mixed findings elucidate the need for further research into whether gender differences exist within adolescent populations, as well as an analytical approach that involves separating youth by age-groups to identify where gender differences may or may not exist.

The ability to attribute mental states to oneself and others (Theory of Mind, ToM) allows for an understanding of the shared human experience [20]. The realisation that other individuals experience and are motivated to avoid suffering theoretically allows for more kind and considerate interaction. Children’s ToM abilities have been linked to friendliness and kind acts [21,22]. For example, Fink et al. [21] found that five- to seven-year-olds with at least one mutual friend significantly outperformed ‘chronically friendless’ peers on both basic and advanced ToM tasks. Related findings support the notion that the ability to understand another’s perspective or mental view has a possibly positive influence on friendship in pre-adolescents, and thus may help protect against loneliness [23,24]. In addition, studies show that children proficient in mindreading show more complex peer interaction skills (e.g., more skilled cooperative communication), as well as increased social motivation (e.g., expressed willingness to build and maintain friendships) [25].

These findings align with social outcomes theory, which suggests that those with more advanced social cognition learn to build and maintain more secure social relationships [26,27]. One possible reason for this may be that those with advanced social cognitive abilities also have increased levels of kindness. So far, studies have explored prosociality as a likely outcome of advanced ToM abilities [28]; however, this study is novel in focusing on whether ToM is specifically linked to kindness. Overall, previous studies have suggested that individuals who score highly on ToM tests perform more prosocial behaviours compared to those who have relatively low scores [28], supporting the view that mindreading ability may be closely related to prosocial characteristics such as kindness and compassion, however, the present study aims to test this hypothesis directly.

Although empirical evidence has demonstrated that ToM capacity can be positively related to benevolent and prosocial acts such as helping and comforting others [27,28]. ToM has also been linked to malevolent and deceptive acts, such as increased bullying behaviours and relational aggression [29,30,31]. Therefore, it is of interest to further explore the interrelations amongst kindness, compassion, kind acts, and ToM, in order to answer the question of whether the ability to understand the minds of others is related to adolescent kindness specifically.

### 1.4. Kindness and Well-Being

As mentioned, acts of kindness have been robustly evidenced as being beneficial to both the recipient and the actor [5,32] in socio-psychological ways. For example, it results in increased happiness, life satisfaction and optimism [5,33,34], as well as social connection and decreased loneliness [35,36] Recently, studies have explored the benefits of kindness in terms of well-being in younger samples, with a recent review [37] demonstrating that children as young as 3–10 years old derive happiness from performing morally good (i.e., kind) behaviours. Similarly, a recent study [38] found that those adolescents who acted kindly reported reduced feelings of loneliness, suggesting that acting kindly has adolescent-specific well-being benefits. Considering that adolescent conceptualisations of happiness evolve to include recognition of social influences [39], it would be valuable to explore how kindness correlates to well-being specifically during adolescence.

### 1.5. The Present Study

The aim of the present cross-sectional, mixed-methods study was to explore the associations between adolescent ToM, kindness, compassion, and well-being. Specifically, we wanted to test the relationship between social cognition (ToM) and kindness in adolescents, as well as the developmental pattern in relationships between kindness and the kind actors’ own social and psychological well-being. We were particularly interested in sampling a broad adolescent sample due to previous studies primarily focusing on pre-adolescents, early adolescents, or adults.

Past studies on kindness and compassion predominantly focus on young or older adults and often neglect adolescents (between 11 and 18 years) (see [5] for a meta-analysis of studies examining the effect of kindness on well-being). Studies on how adolescents express and conceive of kindness and their social experiences are limited [40], often using either quantitative measures only, small-scale focus groups [10], or focusing exclusively on kind acts [11,12]. Hence, this study aims to provide a general exploration of kindness in adolescents, providing a foundation for future research.

Based on studies suggesting that factors related to ToM and prosociality develop during adolescence (such as self-compassion [41,42,43]), we hypothesised that the link between high levels of ToM and high levels of prosociality would differ according to age. More specifically, we expected that the positive relations between ToM and prosociality would be stronger in late adolescence due to age-related decreases in ego-centrism [44,45]. Thus, based on the previous literature, the following research questions and hypotheses were developed:

**RQ1.**  *How do adolescents perceive kindness towards the self and others, in terms of thought, language and action?*

**H1.**  *There will be a difference in kind acts, depending on the relationship to the recipient (self vs. strangers vs. friends).*

**H2.**  *There will be a difference in kind acts, depending on the situational context (school vs. home).*

**RQ2.**  *What are the interrelations among kind thoughts, acts, ToM and psycho-social well-being in adolescents? How do these links differ in terms of age and gender?*

**H3.**  *There will be positive relations among measures of kindness and measures of well-being.*

**H4.**  *The relations between ToM and prosociality will be positive, and increase in strength with age.*

In sum, we expect that adolescent kindness will differ depending on whom they are being kind to and in which situation the act occurs. Secondly, we expect that adolescent kindness will positively impact actors’ social and psychological well-being. Finally, we expect to find that adolescents with high levels of theory of mind will be more prosocial, and that this relationship will report as stronger in older (versus younger) adolescents. We hope that this study will provide a foundation for further, in-depth research into adolescent prosociality, specifically related to kindness.

## 2. Materials and Methods

**Kindness** was measured using six qualitative questions produced for the purposes of the present study. The first three questions asked about kind acts towards strangers, friends and the self (e.g., “*What do you do to act kindly to strangers/self/friends?*”). Participants also provided a definition of kindness, in response to the prompt: “*What does it mean to be kind? Kindness is …*” and provided examples of kindness in a school and home context. For the quantitative analyses, the frequency of kind acts mentioned across questions was taken as a variable: Number of Kind Acts mentioned. Due to the qualitative nature of these questions, we did not quantitatively compare answers to pre-established measures.

Theory of Mind (ToM) was assessed using two well-established and validated measures used with children and adolescents; ‘Silent Films’ (SF) [23] and ‘Strange Stories’ (SS) [46] combined scores. The SF measure involved participants watching six films and interpreting the protagonist’s behaviour in an open-response format (e.g., “*Why do the men hide?*”). Responses were coded on a scale from 0 (*uninterpretable*) to 2 (*pass*), with higher scores indicating more advanced ToM. The SS measure involved participants reading five short stories and interpreting the protagonist’s behaviour in an open-response format. Responses were rated on a three-point scale: 0 (*incorrect*) to 2 (*full and explicit*), with higher scores indicating more advanced ToM. To calculate an overall ToM score for each participant, the sum of their total SF and SS scores was calculated. Combined SF and SS items in the present study showed moderate reliability (*α* = 0.537*).* The SF and SS task battery has previously demonstrated strong reliability and validity and have been used with participants across cultural and age groups [23,47,48].

**Compassion** was measured using the Compassion For Others scale (CS; [49]), which including 16 items, such as: “*I listen patiently when people tell me their problems*”. Participants indicated their agreement with statements on a Likert scale of 1 (*almost never*) to 5 (*almost always*). Higher scores indicated higher levels of compassion for others. Combined compassion items in the present study showed high reliability (*α* = 0.843). The subscale of ‘Kindness’ from this measure was also used, separately, to measure kindness. Combined kindness subscale items in the present study showed high reliability (*α* = 0.767). Previous studies provide evidence for the reliability and validity of the CS scale overall, as well as for its use with adolescents [23,50].

**Psychological Well-being** encompassed two measures: subjective happiness and perceived stress. Due to a low alpha-coefficient when combined, these measures were analysed and discussed independently to preserve reliability.

**Subjective Happiness** was measured by one item taken from the Subjective Happiness Scale (SHS) [51]: “*In general, I consider myself …*”, answered on a scale of (1) *not a very happy person* to (7) *a very happy person*. Higher scores indicated higher levels of subjective happiness. The SHS has been widely used in the previous literature and shows consistent validity for use with adolescents [52,53].

**Perceived Stress** was measured using four items from the Perceived Stress Scale (PSS, shortened edition [54] such as: “*How often have you dealt successfully with irritating life hassles?*”, rated on a scale from (0) *never* to (4) *very often*. PSS total scores were obtained by reversing positively scored items and then summing across scale items. Higher total scores indicated higher levels of stress. Combined stress items in the present sample showed moderate reliability (*α* = 0.686); however, the PSS is well-established as a reliable measure of stress across age-groups [55,56].

**Social Well-being** encompassed three measures: loneliness, aloneliness, and friendship (quality, quantity). Aloneliness items were first reversed, then a mean overall score was calculated for each measure, which combined to form one overall measure of social well-being (*α* = 0.435). Higher scores indicated higher levels of social well-being.

**Loneliness** was measured by items taken from the peer loneliness subscale of the Loneliness and Aloneness Scale for Children and Adolescents (LACA [57]), aimed at measuring quality and quantity of peer friendship. Twelve items were rated on a scale of 1 *(often)* to 4 *(never)*, with lower total scores indicating higher levels of loneliness, suggesting lower levels of social well-being: for example, “*I feel isolated from other people*”. This subscale has been shown to have high concurrent validity with other measures of loneliness in youth, and is recommended as the most accurate measure of social loneliness amongst LACA subscales [58].

**Aloneliness** was measured by The Solitude and Aloneliness Scale (SoIAS [58]). Twelve items were rated from 1 (*strongly disagree*) to 5 (*strongly agree*), with higher overall scores indicating higher levels of aloneliness and therefore lower levels of social well-being: for example, “*I would be happier if I could get more time to myself*”. SoIAS has previously been found to have high internal reliability and construct validity, therefore being suitable for use in adolescent samples [59].

### 2.1. Friendship

(i) **Friendship Quality** was measured using items taken from the emotional support subscale in the Adolescent Friendship Structure Inventory (AFSI [60]). Participants were asked to: “*think of someone who is your number one best friend*”, and rate three items on a scale of 1 *(not true at all)* to 5 *(really true)* for three statements: for example, “*My friend is someone I can talk to about things I’m ashamed of*”. Higher scores indicated higher levels of emotional support from close friends, which indicated high friendship quality and higher social well-being. AFSI has been validated for use in an adolescent sample, showing strong psychometric qualities [60].
(ii) **Friendship Quantity** was measured using four items (AFSI [60]), which asked questions such as “*How often do you make plans to meet with friends?*”. Participants were required to answer from 6 to 8 pre-determined responses. Scores were given to answers, with higher scores indicating high friendship quantity and therefore high social well-being.

### 2.2. Participants and Procedure

The present study formed part of a larger longitudinal study exploring social, emotional and cognitive development in Canadian adolescents. A total of 331 participants were recruited at the time of this study using volunteer sampling. Six participants were excluded due to being of an age outside of 10–18 years (*N* = 3) or missing participant information (*N* = 3). Seven participants identified as a gender category other than ‘male’ or ‘female’ and were therefore excluded from gendered quantitative analyses due to sample sizes that were too low to form a discrete gender category in the analyses. These exclusions led to an adjusted overall *N* = 318 of adolescents aged between 10 and 18 years (Mage = 14.58, *SD* = 2.31).

Participants were grouped into early adolescence (10–13 years, *N* = 138) mid-adolescence (14–16 years, *N* = 109) or late adolescence (17–18 years, *N* = 71). A total of 166 (52.4%) participants identified as female and 145 as male (45.1%). Most participants stated speaking English as a first language (*N* = 227, 71.38%) with 33.33% being White Canadian or European (*N* = 106), 14.78% Black Canadian/Caribbean/African (*N* = 47), 11.01% Asian/Asian Canadian (*N* = 35), 11.64% of mixed ethnicities (*N* = 37), and 5.97% (*N* = 19) from other cultural backgrounds. The remaining participants did not specify cultural background (76.73%, *N* = 244).

Following ethical clearance from the universities and participating schools, participants provided parental as well as verbal assent to participate in the study. Following this, participants completed a self-report questionnaire, administered online via the platform Qualtrics. Any identifying information was anonymised prior to data analysis. After completing the questionnaire, all participants were debriefed and provided with e-gift certificates for their time.

### 2.3. Coding Procedure

Participants completed a series of six open-ended questions about kindness. This included their definitions of kindness (“*What does it mean to be kind? Kindness is* …”), examples of kindness at school or at home, and how they act and speak kindly towards themselves, as well as to strangers or friends. A qualitative content analysis was employed to describe adolescent responses to a series of open-ended questions about kindness based on the procedures outlined by Elo and Kyngäs [61].

The first step of the coding and analysis process included a thorough reading of all responses twice to become familiar with the data and ‘obtain a sense of the whole’ [61]. Following this, we coded the data according to pre-determined categories as per the previous literature. Following the procedure of a previous study on adolescent kindness [11], definitions were inductively coded in terms of positive or negative orientation, as well as in terms of detail. The purpose of this was to help explore developmental trends in adolescent kindness, including how the level of detail and type of definition may change between age-groups. A positive definition was one which described what kindness *is*, and a negative definition was one which described what kindness is *not*. Simple responses were those with a low level of detail or explanation, and complex ones were those with a higher level. Additionally, we coded responses in terms of mental state, that is, whether the response was psychological or purely behavioural in language. For example, “*wash the car*” would be coded as behavioural or action-based with no mention of mental states, whereas “*I care for them and think about their happiness*” would be coded as psychological, which included references to mental states such as thoughts and emotions.

For the second stage of code development, we took an inductive approach. This involved the first author again reading through responses for familiarisation, and from this creating an initial set of categorical codes to describe the themes within the data. From this, the author grouped codes into higher order themes that appropriately characterised the data. The codes were discussed with a research assistant and codes were combined or separated where necessary. This led to six thematic coding lists, one for each open-ended question.

Where coding lists had significant overlap, they were used for multiple questions. For example, we used the same coding list in questions regarding kind acts towards friends and strangers (see Section 3).

To ensure codes were reliable and represented the data, a research assistant unfamiliar with the data was trained in coding for orientation, mental state, complexity and thematic content. A 20% randomly selected subset of responses were then independently coded by this assistant and the first author. Ratings given by each coder were compared and discussed, leading to inter-rater reliability meeting or exceeding 80% agreeance for all questions and codes. Additionally, we chose to analyse only the manifest content of responses in order to not impose any assumptions onto the adolescents’ responses. The final step of inductive analysis involved coding all the data in accordance with the final higher-order categories.

## 3. Results

### 3.1. How Do Adolescents Perceive Kindness Towards the Self and Others, in Terms of Thought, Language and Action?

#### 3.1.1. Adolescent Definitions of Kindness

There were a wide range of responses when defining kindness. Most adolescents defined kindness in positive terms—that is, defining what kindness *is* (88.05%, *N* = 280) as opposed to what kindness *is not* (0.63%, *N* = 2). Thirty-six participants (11.32%) incorporated both positive and negative statements into their definitions. Most adolescents defined kindness as only other-oriented (*N* = 289, 90.88%), with a minority including the self in definitions (*N* = 29, 9.12%). Interestingly, many other-oriented definitions incorporated altruism, for example: being “*selfless*” (17-year-old male) and “*helping somebody out when you don’t benefit from it*” (12-year-old male). With regards to age, late adolescents provided the highest proportion of other-oriented responses (92.96% of late adolescent definitions, *N* = 66), followed by mid-adolescents (91.74%, *N* = 100) and then early adolescents (86.96%, *N* = 120), suggesting a developmental trend in kindness conceptualisation. Females were slightly more likely to give a definition that incorporated the self (10.84% of female definitions, *N* = 18), compared to males (8.28% of male definitions, *N* = 12). A total of 207 definitions were coded as simple (65.09%), compared to 111 coded as complex (34.91%) (see Section 2 for further details). Examples of simple responses included the following: “*helping others*”, “*being nice to yourself and others*”, and “*not being mean*”. Complex responses included a higher level of detail and were more developed, for example:

*“To me, kindness means being compassionate, caring, and considerate towards others. Kindness can take many forms, such as offering help, showing empathy, and even just a simple smile. It’s often said that kindness is the language which the deaf can hear and the blind can see, which I think means that kindness can be understood and appreciated by everyone, regardless of ability or background.”—Boy (14 years)*

The proportion of complex definitions almost doubled between early adolescence (26%) and mid-adolescence (41%); however, this only increased by 2% between mid-adolescence and late adolescence (for late adolescents, 43% of definitions were coded as complex), suggesting that adolescent conceptualisations of kindness show a marked peak in development between 10 and 13 years. In terms of gender differences, females were slightly more likely to give a complex definition (37%) compared to males (32%).

Mental-state coding revealed that 218 participants (68.24%) included psychological (emotional or cognitive) terms into their definitions of kindness: for example, “*having yourself and others at heart*” (14 year old boy), or “*when people think before themselves*” (11 year old female). As age increased, definitions became more psychological, making up 57% of early adolescent definitions, 76% of mid-adolescent definitions, and 87% of late adolescent definitions. Females, overall, provided a higher proportion of psychological definitions (74% of definitions were psychological), compared to males (66%).

#### 3.1.2. Kind Acts

Kind acts towards others were explored using the following six questions: “What do you do you be kind to.” (friends, strangers and self) and “What is an example of kindness you have done at home(/school)?”. Whilst participants were provided with six questions, many participants stated more than six kind acts. Overall, 1723 kind acts were provided by the adolescents, all of which were thematically analysed for content. See Table 1 for an overview of themes and frequencies across acts.

Across 1723 kind acts, the most common theme was helping (*N* = 463), followed by proactive support (*N* = 331), respect (*N* = 318), emotional support (*N* = 200), generosity (*N* = 198), compliments (*N* = 78), motivational acts (*N* = 50), and gratitude (*N* = 31). Participants gave 34 responses stating that they were unsure how to answer. Helping remained the most frequent theme across age and gender groups.

Across kind act questions, responses were coded overall in terms of mental state. A total of 1549 (54.16%) responses were coded as psychological, while 1131 (39.56%) responses were coded as purely behavioural. For 180 of the responses to kind act questions, participants either gave no response or stated that they were unsure on how to answer (6.28%).

### 3.2. H1: There Will Be a Difference in Kind Acts, Depending on the Relationship to the Recipient (Self vs. Strangers vs. Friends)

Participants reported 490 kind acts towards friends, 792 towards strangers, and 513 towards the self. Inductive thematic analysis revealed the same emergent themes of kindness in kind acts towards strangers and friends, but distinct themes for kindness towards the self. The most frequent themes for each recipient group were as follows: emotional support (friends; 23.67%), respect (strangers; 27.65%) and mental health (self; 30.41%). See Table 2 for a complete overview.

### 3.3. H2: There Will Be a Difference in Kind Acts, Depending on the Situational Context (School vs. Home)

Participants reported 352 examples of kindness in a home context and 349 in a school context. Inductive thematic analysis revealed the same emergent themes of kindness in kind acts in both situational contexts; however, the most commonly reported themes differed between contexts. See Table 3 for a complete overview.

When asked to give an example of kindness in a school environment, most responses reflected a theme of proactive support (30.37%) or helping (27.80%). Unsurprisingly, most instances of helping responses involved helping somebody academically, for example: “*helping somebody to study and do homework*” (16-year-old female). In a school context, examples of proactive support typically involved intervening with bullying or peer loneliness (“*Stood up for a student being bullied*” (16-year-old male); “*I spent time with someone who is usually alone at school*” (11-year-old female).

More than half of the examples of kindness in a home environment reflected the theme of helping (51.14%), followed by proactive support (24.72%). In this context, kind helping actions were more centred around practical chores, for example: “*I have assisted my mum in packing the toiletries (…)*” (18-year-old female), or “*Helping my parents with house chores*” (11-year-old female).

### 3.4. Quantitative Analyses

Inferential data analysis was performed using IBM SPSS for Mac Version 28. Prior to analysis, all variables were subject to skewedness tests of normality and central tendencies were calculated. Most variables were normally distributed; however, Theory of Mind showed a highly negative skewed distribution (−9.963), suggesting that most individuals scored highly on this measure. No outliers were removed from the analysis due to the aim of analysing true ToM abilities in adolescence. As a result, not all variables had a non-normal distribution, and therefore the assumptions of Pearson’s correlational test were not met. For this reason, and as most data was ordinal, Spearman’s Rho correlational analyses were used to quantitatively measure the interrelations between kindness, ToM and psycho-social well-being [51]. Participants who had not completed at least 50% of scales involved in ‘Social Well-being’ were excluded from analysis involving this measure (*N* = 3).

#### 3.4.1. What Are the Interrelations Among Kind Thoughts, Acts, ToM and Psycho-Social Well-Being in Adolescents? How Do These Links Differ in Terms of Age and Gender?

To investigate inter-relations amongst the variables of ToM, kindness and well-being, Spearman’s Rho correlations were conducted. Correlations were conducted firstly on all participants to identify overall relations, and secondly on the dataset separated by age and gender categories to investigate group differences. To test whether correlational differences between age categories were significant, Fishers R-Z transformations were performed. See Table 4 for a complete overview of the correlational results and descriptive statistics.

#### 3.4.2. H3: There Will Be Positive Relations Among Measures of Kindness and Measures of Well-Being

Our analyses revealed highly significant correlations between measures of kindness towards others and well-being. There were significant positive correlations between Kindness and Social Well-being (*r* = 0.271, *p* < 0.001), Number of Kind Acts and Social Well-being (*r* = 0.116, *p* = 0.041), Number of Kind Acts and Compassion (*r* = 0.256, *p* < 0.001) and Compassion and Social Well-being (*r* = 0.291, *p* < 0.001). These results indicate that kind and compassionate adolescents have improved social well-being, when conceptualised as decreased loneliness and aloneliness, and increased friendship quality and quantity (see the Discussion section for further elaboration on this point). No significant correlations were found between kindness and compassion, or between kindness, happiness, or stress. However, positive relations were found between social well-being and happiness (r = 0.345, *p* < 0.001) and negative relations were found between social well-being and perceived stress (*r* = −0.111, *p* = 0.049).

When separated by gender, most relations remained significant. However, the relations between Number of Kind Acts and Social Well-being became non-significant for both gender categories (males: *r* = 0.071, *p* = 0.405; females: *r* = 0.145, *p* = 0.063). Additionally, the negative relation between perceived stress and social well-being was only significant for females (*r* = −0.198, *p* = 0.011), and not for males (*r* = −0.029, *p* = 0.731). These results indicate that social well-being relates to reduced stress levels in female adolescents only.

#### 3.4.3. H4: The Relations Between ToM and Prosociality Will Be Positive and Increase in Strength with Age

The relation between ToM and compassion was non-significant in early adolescents; however, results showed a significant positive correlation in mid-adolescents (*r* = 0.245, *p* = 0.011) and in late adolescents (*r* = 0.465, *p* < 0.001). To investigate H3, a two-tailed Fishers R-Z transformation was conducted, and results showed a significant age group difference for this correlation between early adolescents and late adolescents (*z* = −2.35, *p* = 0.01).

The same pattern was present for the relations between Kindness and ToM, which were non-significant in early adolescents; however, there was a significant positive relation in mid-adolescents (*r* = 0.213, *p* = 0.028) and in late adolescents a (*r* = 0.498, *p* < 0.001). A Fishers R-Z transformation confirmed a significant difference between early and late adolescent group co-efficients (*z* = 0.058, *p* < 0.01). Overall, results indicate that there is a significant increase in the strength of relation between Compassion and ToM and between Kindness and ToM from early to late adolescence.

In all age categories, there was a significant positive correlation between ToM and number of kind acts, as well as between ToM and social well-being (in terms of friendship quality, quantity, reduced loneliness and aloneliness). A Fishers R-Z transformation found the difference between early adolescents and late adolescents to be non-significant for both correlations, suggesting the relations between ToM and social well-being and between ToM and number of kind acts given do not significantly change between early and late adolescence, remaining stable. Table 5 provides an overview of the correlations between prosociality variables (compassion, kindness), psycho-social well-being variables and ToM, separated by age category.

## 4. Discussion

This cross-sectional, mixed-methods study explored how adolescents conceptualise kindness in different relational and situational contexts, as well as investigated the inter-relations among mentalization skills (ToM), kindness, compassion and well-being. The purpose of this study was to test the relationship between social cognition (ToM) and kindness in adolescents, as well as the relationships between kindness and the kind actors’ own social and psychological well-being. We were particularly interested in the adolescent-specific developmental trajectory of kindness. Results will be outlined below, guided by our research questions, followed by an outline of the study’s limitations and implications.

### 4.1. How Do Adolescents Perceive Kindness Towards the Self and Others, in Terms of Thought, Language and Action?

Although research regarding kindness has been gaining interest in recent years, studies have prioritised pre-adolescent and adult samples, leading to a paucity in research examining the experiences of adolescent youth [5]. Hence, the present study examined kindness in adolescents across a broad age range (10–18 years) using qualitative content analysis of responses to six open-ended questions.

We found evidence for a developmental trajectory in kindness conceptualisation, specifically in that definitions of kindness increased in complexity and mental-state reference with age, suggesting that late adolescents had a more developed understanding of what it means to be kind compared to younger adolescents. This may be due to an increasingly complex social-cognitive system, or simply to increased lived experience of kindness. This trajectory aligns with progressive theories of moral development, such as Eisenberg and Kohlbergs’ theories of moral reasoning [62,63].

We found that adolescents typically spoke of kindness as an other-oriented phenomenon rather than as something that is directed towards the self. In line with Eisenberg’s theory, the tendency to define kindness in other-oriented terms became more likely with age, suggesting that late adolescents move towards a less self-focused conceptualisation of what it means to be kind compared to early adolescents. This supports previous findings regarding egocentrism in youth, which suggest that a heightened sense of self-importance is characteristic of adolescence, decreasing as individuals move towards early adulthood [44,45], and that this influences prosocial reasoning in terms of moving away from hedonistic and approval-focused forms of prosociality (self-serving kindness; [63]).

Overall, we found that the emergent themes fit with the few existing studies exploring adolescent kindness [9,10], supporting findings that adolescents express kindness through acts of respect, helping, emotional support, generosity, compliments and proactive support. In addition, the results showed that the most common type of kind act performed by adolescents differed based on relational and situational context, supporting H1 and H2.

#### 4.1.1. H1. There Will Be a Difference in How Kindness Is Acted on, Depending on the Relationship to the Recipient (Self vs. Strangers vs. Friends)

A novel and unique finding of this study was that adolescents most frequently showed themes of ‘respect’ in kindness towards strangers, but showed themes of ‘emotional support’ towards friends. Such findings support our hypothesis that there would be relational influences on how adolescents enact kindness towards others. These results suggest that behavioural, non-verbal kind acts (such as acts of respect) are a common means for adolescents to communicate prosocially with those that they are not comfortable enough with to provide emotional or psychological support to.

Our study showed that adolescents were much more likely to be unsure of how to act kindly towards strangers compared to friends. This finding can be understood in terms of the Social Exchange Theory [64], which posits that we decide to behave based on a rational cost-benefit analysis. It is possible that uncertainty comes from a lack of predictability around the risks and benefits of acting kindly towards somebody with whom there are not established social norms and expectations with. Reduced familiarity with strangers (versus those relationally close to us) may lead adolescents to struggle to predict how behaviours will be received, causing hesitation or confusion regarding how to behave. Additionally, this finding can be understood in terms of Self-Determination Theory [65], which posits that an individual’s sense of relatedness is a key factor in deciding how to behave. Our findings on the importance of the actor–recipient relationship for adolescent kindness has important implications for classroom kindness towards socially-isolated pupils.

A further finding of the present study was that adolescents spoke of self-kindness in distinct thematic ways compared to other-oriented kindness. Taken with the finding that most adolescents defined kindness as other-oriented, the present study suggests that adolescents conceive of kindness and self-kindness as distinct phenomena, providing a basis for future research.

#### 4.1.2. H2. There Will Be a Difference in How Kindness Is Acted on, Depending on Situational Context (School vs. Home)

The present study also found partial support for situational variation in adolescent kindness. When reporting kindness in a home context, over half of all examples given involved helping behaviours, typically with household chores. Other present but less frequent expressions of kindness included expressions of gratitude and giving compliments. These findings suggest that to develop adolescent kindness in a home environment, it would be valuable to facilitate opportunities for helping behaviours (particularly chores-based behaviours), as well as education and scaffolded support with less common, verbal kindness acts such as compliments and expressions of gratitude.

With regards to school-based kindness, we found that adolescents most frequently reported proactive support, such as reaching out to an isolated or victimised peer. Such findings are in line with previous studies examining themes of school-context kindness in children [66]. Compared to kindness at home, helping behaviours made up a relatively smaller, but still notable, number of examples given. Similar to with home-based kindness, a present but small proportion of responses mentioned expressing kindness verbally, for example through compliments or gratitude. For educational environments to encourage kindness with breadth and depth, it would be beneficial to facilitate opportunities for typical kind behaviours (such as minimal involvement in group tasks, to encourage proactivity), as well as to scaffold less common kind behaviours (such as providing opportunities to learn about verbal expressions of kindness). This study provides novel insights into how it may be valuable for kindness-facilitators to consider which themes of kindness are most accessible to adolescents given the situational context.

### 4.2. What Are the Inter-Relations Among Kind Thoughts, Acts, ToM and Psycho-Social Well-Being in Adolescents? How Do These Links Differ in Terms of Age and Gender?

A main goal of our study was to identify the relations among kindness, compassion, ToM and well-being measures. The positive link between mentalization abilities and kindness supports studies that show those who experience a high need for cognition and complexity also are less likely to engage in aggressive acts [67]. Results also showed positive relations among kindness, compassion for others, and overall social well-being, although no links to perceived stress and happiness. These results suggest that the adolescent-specific well-being benefits of kindness are social and may be more related to aspects of friendships and peer relationships, rather than psychological aspects of well-being such as how one copes with stress and feelings of happiness. Our results contrast previous research with adults that indicate well-being benefits of kindness are multifaceted (i.e., including issues of mental health as well as social relationship factors) [5,33,34], raising important questions about which population may benefit from psychological well-being focused kindness interventions. Such age-related differences in findings suggests the need for future research on links between mentalization, prosociality, and emotional and social well-being across the lifespan.

#### 4.2.1. H3. There Will Be Positive Relations Among Measures of Kindness and Measures of Well-Being

Results showed partial support for the hypothesis that there would be positive relations among measures of kindness and well-being. Specifically, kind, compassionate adolescents who could provide a high number of kind act examples had higher levels of social well-being; that is, they were less lonely and alone, with higher levels of friendship quality and quantity. Again, this finding can be viewed through the lens of Social Exchange Theory [64], which posits that behaviour is guided by a rational weighing of the costs and perceived benefits of a proposed action. If an individual feels that the perceived benefits outweigh the costs of acting kindly, then they will continue this action. Through acting kindly, our findings suggest that the adolescent receives social benefits such as increased friendship quality and quantity, as well as reduced feelings of loneliness and solitude. Although the actor may initially act for a distinct reason (such as empathy motivated by ToM, or relatedness), the benefits to social well-being act as a driver to act kindly again. This may lead to a reciprocal dynamic, whereby acting kindly improves social-well-being and improved social well-being increases the propensity to act kindly. Future research should investigate this further by elucidating the directionality of the relationship between kindness and social well-being in adolescent populations.

This builds upon previous findings reporting a link between ToM and social well-being measures in pre-adolescent children [21,22], indicating that kindness could be a valuable intervention for loneliness and social isolation in adolescents. This implication was further strengthened by the finding of a link between social well-being and multiple measures of prosociality (kindness, compassion, and number of kind examples given).

Interestingly, we did not find significant relations between kindness, compassion or number of kind act examples given and measures of psychological well-being, meaning that prosocial adolescents did not necessarily have reduced stress or increased happiness, as previous non-adolescent studies have suggested [5,33,34]. The absence of this significant finding contrasts with previous studies that suggest that adult kindness positively impacts psychological well-being [5], suggesting that the benefits of kindness to the actor may be subject to the age-group of the individual. This requires further research to determine whether the relationship between well-being and kindness is truly different in adults and adolescents as this result initially suggests.

However, we found that adolescents with high levels of social well-being (e.g., high quality friendships), also had higher levels of psychological well-being (e.g., reduced perceived stress and increased happiness). These findings suggest that although acting kindly may not impact the adolescent’s stress and happiness levels directly, it does link to their social well-being, which in turn correlates with psychological well-being. Therefore, it would be of value for future research to explore the psychological benefits of kindness and compassion in terms of well-being in an indirect sense, through measures of social well-being.

#### 4.2.2. H4. The Relations Between ToM and Prosociality Will Be Positive and Increase in Strength with Age

Our findings supported H4, which posited that the relations between ToM and prosociality would be different in early as compared to late adolescence. Specifically, we found that the positive relationship between ToM and compassion, as well as that between ToM and kindness, increased in strength between early and late adolescence. This suggests that 17–18 year olds with advanced ToM abilities are more likely to be kind and compassionate compared to younger adolescents. This finding may be explained by Self-Determination Theory [65], which poses that the development of perceived competence can intrinsically motivate us to act; as the ability to perspective-take develops, adolescents may feel more competent to act kindly in ways that they understand will be valued by the recipient. Future studies may wish to further explore the role of perceived competence in adolescent kindness.

Additionally, previous studies have demonstrated that as adolescents approach early adulthood, they have a more developed and integrated sense of self [68]. Taken with these findings, it may be the case that older adolescents have developed and internalised personal values such as kindness, therefore leading to a stronger association between the understanding of others’ minds and the enactment of kindness and compassion in this age group compared to early adolescents. Social-psychological research in adolescence also shows that late adolescents (compared to early adolescents) may be less concerned with others’ perspectives about themselves and with gaining social approval [69]; therefore, those with higher mindreading abilities in late adolescence (vs. early adolescence) may be more likely to act in kind ways, due to a reduced consideration of the social costs of kindness (e.g., social rejection from peers for acting kindly towards a bullying victim).

### 4.3. Implications, Limitations and Future Directions

Although the present study offers valuable insights into adolescent kindness and correlates, there are a number of limitations to address. Firstly, the sample in the present study were Canadian adolescents, who mostly spoke English as a first language. Therefore, generalised conclusions about kindness in adolescents from this study should be approached with caution, particularly due to potential cultural differences in social norms. For example, within individualistic cultures (such as those of North America), kindness has been linked to more self-serving motivations compared to motivations within collectivist cultures where kindness is often altruistic [70,71,72]. Cross-cultural differences in motivations such as this indicate that adolescent kindness may be conceived of, and acted on, differently outside of our specific sample. Additionally, it is possible that the relations between kindness and well-being may vary as a function of cultural context and social motivations. The present study should be taken as representative of Canadian adolescents, and caution should be taken when generalising outside of this context.

Secondly, we explored psychological well-being in terms of reduced stress and increased happiness. It would be beneficial for future studies to consider other dimensions of psychological well-being in adolescent samples to further investigate the benefits of kindness in terms of well-being. Considering that our findings contradict studies into the positive impact of prosociality on psychological well-being in non-adolescent samples [5,33,34], it is important for future studies to further investigate whether kindness exclusively supports social well-being in adolescents. It may also be beneficial for follow-up studies to exercise a range of sampling and experimental methods to triangulate any potential biases as a result of self-reported data or volunteer sampling.

A further point to note is that skewness analyses indicated ceiling effects in ToM measurements. Therefore, it would be beneficial for future studies to consider scaling the difficulty of ToM measures when used with older samples to avoid potential ceiling effects, as well as further meaningfully investigate advanced ToM abilities in late adolescents.

The present study provides a novel insight into how kindness develops across the spectrum of adolescence and illustrates how Theory of Mind and mental-state talk increasingly influence adolescent kindness with age. Future studies can further explore other cognitive and emotional skills relating to ToM, such as working memory, emotional and cognitive regulation, affective empathy, person perception and cognitive flexibility, to further explore the role of social cognition in adolescent prosociality. In addition, it would be valuable for future research to investigate in a variety of cultural contexts, as well as to consider additional dimensions of psychological well-being in relation to kindness. A final direction for future research may be to further investigate the relations between constructs through more advanced statistical modelling, such as mediating or moderating effect analyses, to further elucidate the type of relationship between variables.

Despite its limitations, the present study holds practical implications for educational programs that may promote adolescent kindness and compassion across recipients and situations. First, the present study offers insights into adolescent well-being initiatives and supports connections among kindness, compassion and social well-being in this age group. Our findings suggest that it would be beneficial for kindness interventions to include training of ToM given the strong positive relationships between kindness, compassion and ToM. Understanding kindness across relational and situational contexts provides a direction for parental and educational facilitators, in that accessible forms of kindness (e.g., helping in a home context) should be given the opportunity, and that less common forms of kindness (e.g., verbal kindness) should be scaffolded and supported across contexts.

Thus, this cross-sectional, mixed-methods study shows that adolescents have a diverse understanding of kindness and that this plays a role in the way they think and feel about themselves and others. The present findings may encourage future research aiming to inform empirically based intervention programs that aim to enhance adolescent well-being through nurturing a kind youth.

## Figures and Tables

**Table 1:** Frequencies of kind act themes in adolescents, by age and gender.

Age											
Early Adolescence				Mid-Adolescence				Late Adolescence			
(*N* = 138)				(*N* = 109)				(*N* = 71)			
Male		Female		Male		Female		Male		Female	
(*N* = 63)		(*N* = 72)		(*N* = 45)		(*N* = 62)		(*N* = 37)		(*N* = 32)	
Help.	104 *	Help.	94 *	Help.	66 *	Help.	88 *	Help.	74 *	Help.	53 *
Gener.	35	Gener.	49	Gener.	29	Gener.	42	Gener.	30	Gener.	22
Resp.	59	Resp.	80	Resp.	53	Resp.	59	Resp.	39	Resp.	36
Comp.	10	Comp.	24	Comp.	6	Comp.	25	Comp.	6	Comp.	10
Emot.	31	Emot.	44	Emot.	28	Emot.	43	Emot.	28	Emot.	27
Pro.S.	61	Pro.S.	76	Pro.S.	48	Pro.S.	79	Pro.S.	34	Pro.S.	39
Gratitu.	2	Gratitu.	8	Gratitu.	4	Gratitu.	10	Gratitu.	6	Gratitu.	2
Motiv.	5	Motiv.	8	Motiv.	11	Motiv.	9	Motiv.	13	Motiv.	5
Unsure	10	Unsure	7	Unsure	6	Unsure	8	Unsure	3	Unsure	1
Total	317	390		251		363		233		195	

*Note.* Values represent number of acts mentioned. * refers to the most frequent theme for each category. Help. = helping; Gener. = generosity, Resp. = respect, Comp. = Complimenting, Emot. = emotional support, Pro.S. = proactive support, Gratitu. = gratitude, Motiv. = motivational.

**Table 2:** Frequencies of kind act themes in adolescents, by relationship to recipient.

Friend		Stranger		Self	
Helping	107 (21.84%)	Helping	82 (10.45%)	Digital.	5 (0.97%)
Generosity	72 (14.69%)	Generosity	24 (3.03%)	Physical App.	14 (2.7%)
Respect	47 (9.59%)	Respect	219 * (27.65%)	Phys. Health	68 (13.26%)
Compliment	28 (5.7%)	Compliment	16 (2.02%)	Mental Health	156 * (30.41%)
Emotional Sup.	116 * (23.67%)	Emotional Sup.	24 (3.03%)	Self-Motiv.	44 (8.58%)
Proactive Sup.	85 (17.35%)	Proactive Sup.	49 (6.19%)	Academic	9 (1.75%)
Gratitude	9 (1.84%)	Gratitude	5 (0.63%)	Consumerism	38 (7.41%)
Motivational	21 (4.29%)	Motivational	3 (0.38%)	Morality	14 (2.73%)
Unsure	5 (1.02%)	Unsure	21 (2.65%)	Prosocial	20 (3.90%)
				Self-Care	55 (10.72%)
				Solitude	25 (4.87%)
				Hobbies	53 (10.33%)
				Unsure	12 (2.34%)
Total	490	792		513	

*Note.* All values represent number of acts mentioned across participants. * refers to most frequent theme. Emotional Sup. = Emotional Support; Proactive Sup. = Proactive Support; Physical App. = Physical Appearance; Phys. Health = Physical Health; Self-Motiv. = Self-Motivational.

**Table 3:** Frequencies for kind act themes in adolescents by situational context.

	School	Home
Helping	97 (27.80%)	180 * (51.14%)
Generosity	64 (18.34%)	42 (11.93%)
Respect	34 (9.74%)	12 (3.41%)
Compliment	12 (3.44%)	2 (0.57%)
Emotional Sup.	27 (7.7%)	15 (4.26%)
Proactive Sup.	106 * (30.37%)	87 (24.72%)
Gratitude	0 (0%)	5 (1.42%)
Motivational	4 (1.15%)	5 (1.42%)
Unsure	5 (1.43%)	4 (1.14%)
Total	349	352

*Note.* All values represent number of acts mentioned across participants. * refers to most frequent category. Emotional Sup. = Emotional Support; Proactive Sup. = Proactive Support.

**Table 4:** Descriptive statistics and Spearman’s Rho correlational results for measures of kindness and psycho-social well-being.

Variable	1	2	3	4	5	6	7
1. Compassion	1	0.256 **	0.291 **	0.022	0.050	0.822 **	0.247 **
2. No. Kind Acts	0.256 **	1	0.116 *	0.103	−0.020	0.211 **	0.315 **
3. Social Well-being	0.291 **	0.116 *	1	−0.111 *	0.345 **	0.271 **	0.188 **
4. Stress	0.022	0.103	−0.111 *	1	−0.212 **	0.041	0.076
5. Subjective Happin.	0.050	−0.020	0.345 **	−0.212 **	1	0.104	−0.111
6. Kindness	0.822 **	0.211 **	0.271 **	0.041	0.104	1	0.204 **
7. ToM	0.247 **	0.315 **	0.188 **	0.076	−0.111	0.204 **	1
Skewedness	−0.035	0.802	−0.438	−0.258	−0.878	−0.963	−9.963
Mean Score	3.95	6.90	12.82	2.77	5.41	4.14	12.25
Std. Deviation	0.542	2.712	2.295	0.451	1.17	0.687	7.230
N	313	318	312	313	315	313	313

*Note.* *. *p* < 0.05 (two-tailed). **. *p* < 0.01 (two tailed). Subjective Happin. = Subjective Happiness; No. Kind Acts = Number of Kind Acts.

**Table 5:** Descriptive statistics and Spearman’s Rho correlational results for ToM and measures of kindness or psycho-social well-being.

	Early Adolescence	Mid Adolescence	Late Adolescence
	(10–13, *N* = 138)	(14–16, *N* = 109)	(17–18, *N* = 71)
Variable	ToM	ToM	ToM
1. Compassion	0.152	0.245 *	0.464 **
3. No. Kind Acts	0.295 **	0.289 **	0.267 *
4. Social Well-Being	0.199 *	0.170	0.243 *
5. Stress	0.038	0.017	0.142
6. Subjective Happin.	−0.013	−0.053	−0.291 *
8. Kindness	0.058	0.213 *	0.496 **

*Note.* *. *p* < 0.05 (two-tailed). **. *p* < 0.01 (two tailed). Subjective Happin. = Subjective Happiness; No. Kind Acts = Number of Kind Acts.

## Data Availability

The data presented in this study are available on request from the corresponding author due to ethical and confidentiality reasons.
